# Colonic Granular Cell Tumor: An Endoscopic and Histopathologic Review with Case Illustration

**DOI:** 10.7759/cureus.2015

**Published:** 2018-01-02

**Authors:** Daryl Ramai, Jonathan Lai, Kinesh Changela, Sury Anand

**Affiliations:** 1 Division of Gastroenterology and Hepatology, Academic Affiliate of the Icahn School of Medicine, Clinical Affiliate of the Mount Sinai Hospital; 2 School of Medicine, St. George's University, Grenada, West Indies; 3 Division of Gastroenterology, NYU Langone Hospital, 150 55th St Brooklyn, Ny, 11220.

**Keywords:** granular cell tumor, colon, gastrointestinal tract, schwann cells

## Abstract

Granular cell tumors (GCTs) are rare and benign tumors that can occur at any anatomical site. GCTs are thought to originate from nerve cells, particularly Schwann cells. Their name derives from the fact that an accumulation of cytoplasmic lysosomes imparts the tumor with a granular appearance. They are most commonly observed in the oral cavity, skin and subcutaneous tissue, breast, and respiratory tract. Granular cell tumors rarely affect the gastrointestinal tract. We report a 58-year-old female with a past medical history of hypertension, mitral valve prolapse, and depression who presented for surveillance colonoscopy. A single firm sessile polypoid lesion, with overlying pale tan color mucosa, measuring approximately 1 to 1.5 cm, was found in the ascending colon. Biopsy of the nodule followed by histopathology was positive for S100 and CD68, but negative for AE1/AE3, CD117, smooth muscle actin, and desmin, consistent with the diagnosis of GCT. We review the clinicopathologic features of GCTs.

## Introduction

Granular cell tumors (GCTs) are rare and benign tumors that can occur along any anatomical site. GCTs are thought to originate from nerve cells, particularly Schwann cells [1]. Their name derives from the fact that an accumulation of cytoplasmic lysosomes imparts the tumor with a granular appearance. They are most commonly found in the oral cavity (40%), skin and subcutaneous tissue (30%), breast (15%), and respiratory tract (15%). Gastrointestinal GCTs are rare (8%), and predominantly affect the esophagus, followed by the stomach and duodenum [2]. Of all colonic GCTs, approximately 86% arise in the ascending colon, with GCTs of the rectum being the rarest [2]. Though GCTs can present at any age, its peak incidence occurs in the fourth and sixth decades of life, with a slight female predilection.

## Case presentation

**Figure 1:**
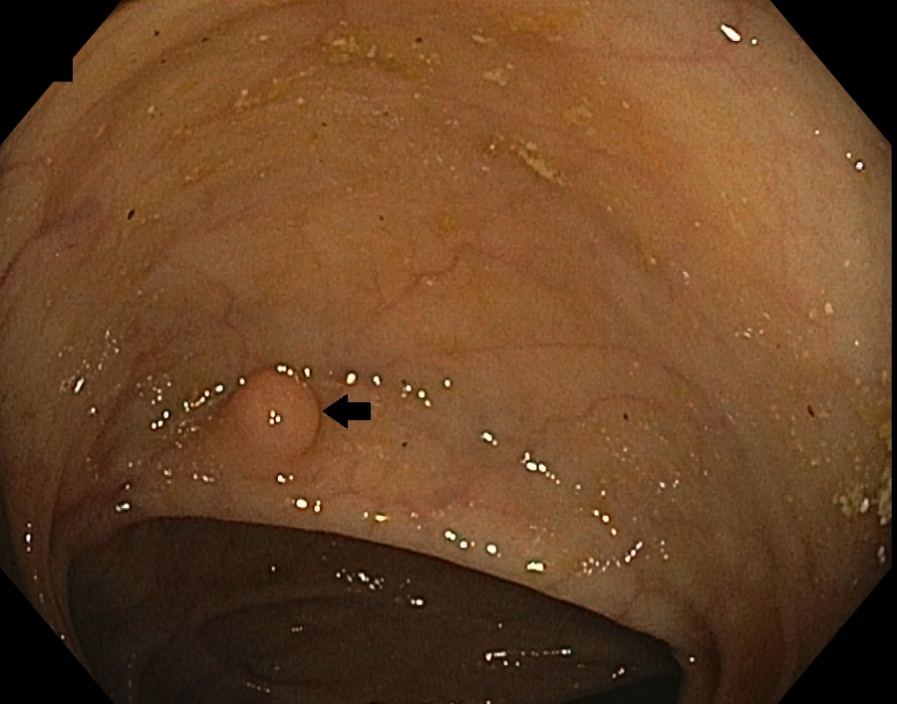
Solitary polyp, measuring 1 to 2 cm and covered by normal-appearing mucosa, was found in the ascending colon

**Figure 2:**
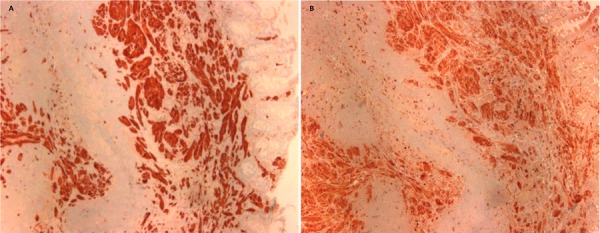
Histopathology of nodule A) Tumor cells showing S100 cytoplasmic immunoreactivity (x40); B) Tumor cells with CD68 immunoreactivity (x40)

A 58-year-old African-American female with a past medical history of hypertension, mitral valve prolapse, and depression presented for surveillance colonoscopy. Vital signs showed the heart rate at 72 bpm, temperature 98.3°F, respiratory rate 14 bpm, and blood pressure 134/73 mmHg. Her physical exam was unremarkable, and she denied alcohol consumption, smoking, or drug use. Laboratory findings showed hemoglobin 11.9 g/dL, hematocrit 38%, white blood count 4.6 x 103 K/µL, and platelet 136 x 103 K/µL. Diagnostic colonoscopy revealed a single firm sessile polypoid lesion with overlying pale tan color mucosa, measuring approximately 1 to 1.5 cm in the ascending colon (Figure 1). The polyp was completely removed by hot snare cautery polypectomy. Histopathology was positive for S100 and CD68 but negative for AE1/AE3, CD117, smooth muscle actin, and desmin, consistent with the diagnosis of GCT (Figure 2).

## Discussion

Granular cell tumor (GCT), also known as granular cell myoblastoma or Abrikossof's tumor, is a benign proliferation of Schwan cells of soft tissues [3]. It was first described by the Russian pathologist, Alexei Ivanovich Abrikossoff, in 1926 [3]. GCTs were initially thought to be myogenic tumors; however, studies have favored a Schwann cell origin. While GCTs may occur at any anatomical location, gastrointestinal tract GCTs are rare. Most cases involve the skin, subcutaneous tissue, and oral cavity. In the GI tract, GCTs are more commonly found in the esophagus, while the cecum and rectum are rarely affected [4].

Gastrointestinal GCTs are typically solitary and represent an incidental finding on screening colonoscopy. Additionally, these polyps are painless, nonulcerated, and firm. Although most case reports highlight that GCTs are benign neoplasms, some cases of malignancy have been reported in the literature, representing less than 2% of all GCTs [5-6]. Additionally, GCTs must be differentiated from other neoplasms, including stromal, carcinoid, and smooth muscle tumors. To this end, GCTs are rarely diagnosed based on macroscopic or endoscopic appearance, particularly due to its small size and shape, resembling a diminutive polyp [6]. In the present case, it was difficult to suspect GCT as the endoscopic features of the tumor resembled those of a small sessile polyp. We performed a one-stage endoscopic snare polypectomy during the screening colonoscopy.

The diagnosis of GCT is confirmed by histopathology and includes (1) plump histiocyte-like, bland-looking neoplastic cells with abundant granular eosinophilic cytoplasms containing acidophilic, periodic acid Schiff (PAS) stain-positive, diastase-resistant granules, (2) small, uniform nuclei in which mitotic figures are absent, and (3) neural markers, including S-100 protein or neuron-specific enolase (NSE), expressed uniformly [7-9]. In the present case, histological findings on the resected specimen were consistent with the diagnosis of GCT. Morphological criteria for malignant GCT include (1) spindling of tumor cells, (2) increased nuclear to cytoplasmic ratio, (3) pleomorphism, (4) necrosis, (5) vesicular nuclei with large nucleoli, and (6) increased mitotic activity (> 2 mitoses per 10 high-powered fields). Fanburg-Smith, et al. reported that if three of the six morphological criteria listed above were present, the lesion was most likely malignant [10].

Malignancy has been found to correlate with tumor size. Approximately more than 60% of metastatic GCTs were larger than 4 cm in diameter [6, 10]. However, in most colonic GCTs, the tumor size was less than 2 cm and the tumor was well separated from the muscularis propia. Since this tumor is considered to be usually benign, endoscopic resection has become the gold standard in the management of gastrointestinal GCTs.

## Conclusions

We report a case of GCT located in the ascending colon. The tumor was removed by endoscopic resection, and the patient was discharged without any complications. Clinicians should bear in mind the differential diagnosis of GCT when presented with submucosal colonic polyps. Additionally, endoscopists should be aware of the pathological characteristics associated with malignant GCTs in order to provide early diagnosis and treatment.
